# The Role of Apparent Diffusion Coefficient in the Differentiation between Cerebellar Medulloblastoma and Brainstem Glioma

**DOI:** 10.3390/neurolint12030009

**Published:** 2020-10-29

**Authors:** Pham Minh Thong, Nguyen Minh Duc

**Affiliations:** 1 Department of Radiology, Hanoi Medical University, Ha Noi 100000, Vietnam; phamminhthong@hmu.edu.vn or; 2 Department of Radiology, Pham Ngoc Thach University of Medicine, Ho Chi Minh City 700000, Vietnam; 3 Department of Radiology, Children’s Hospital 02, Ho Chi Minh City 700000, Vietnam

**Keywords:** cerebellar medulloblastoma, brainstem glioma, magnetic resonance imaging, apparent diffusion coefficient

## Abstract

For certain clinical circumstances, the differentiation between cerebellar medulloblastoma and brainstem glioma is essential. We aimed to evaluate the role played by the apparent diffusion coefficient (ADC) values in the differentiation between cerebellar medulloblastomas and brainstem gliomas in children. The institutional review board approved this prospective study. Brain magnetic resonance imaging (MRI), including diffusion-weighted imaging (DWI) and ADC, was assessed in 32 patients (median age: 7.0 years), divided into two groups, a medulloblastoma group (group 1, *n* = 22) and a brainstem glioma group (group 2, *n* = 10). The Mann–Whitney U test was utilized to compare tumor ADCmax, ADCmin, ADCmean, and ADCsd values, and their ratios with the parenchyma values between the two groups. Receiver operating characteristic (ROC) curve analysis and the Youden index were used to calculate the cut-off value, along with the area under the curve (AUC), sensitivity, and specificity. The median ADCmax, ADCmin, and ADCmean values were significantly higher in group 2 than in group 1 (*p* < 0.05). The median ratios of ADCmin and ADCmean to the parenchyma were significantly higher in group 2 than in group 1 (*p* < 0.05). The ROC analysis showed that the AUC for the ADCmean ratio was the highest among these parameters, at 98.2%. The ADCmean tumor to parenchyma ratio was a significant and effective parameter for the differentiation between pediatric medulloblastomas and brainstem gliomas.

## 1. Introduction

Brain tumors, including supratentorial and infratentorial tumors, are the second-most common malignancies in children, after acute leukemia. The infratentorial region is the primary site for approximately 60% of brain tumors in children. Brainstem glioma is a common tumor in the brainstem area, whereas medulloblastoma is common in the cerebellar region [1,2].

Several studies have reported that some medulloblastomas can infiltrate the brainstem or originate in the brainstem, which can cause misdiagnosis with brainstem glioma [1,3,4]. In addition, after radiotherapy treatments for medulloblastomas, many cases have been reported where the appearance of brainstem glioma has resulted in confusion about whether to diagnose the recurrence of medulloblastoma or a new brainstem glioma [5,6]. The appropriate treatments and prognoses for brainstem glioma and medulloblastoma are completely different; therefore, the diagnostic distinction between these two types of tumors is essential for proper treatment planning and better outcomes [1,2,3,4,5,6].

Magnetic resonance imaging (MRI) has been widely recognized as the best method for assessing pediatric brain tumors because it is completely non-invasive and does not expose the patient to radiation. Although previous studies have evaluated the role of diffusion-weighted imaging (DWI), which is used to assess water molecular motion in tissues, during the differentiation of posterior fossa tumors in children, only a few studies have assessed DWI-MRI during the differentiation of medulloblastoma from brainstem glioma [7,8,9,10,11,12]. Therefore, this study aimed to assess the role of the apparent diffusion coefficient (ADC) during the differentiation between cerebellar medulloblastoma and brainstem glioma.

## 2. Materials and Methods

### 2.1. Patients

The Institutional Review Board of Children’s Hospital 02 approved this prospective study (Ref: 352/NĐ2-CĐT dated 13 March 2020). Informed consent was received from all patients’ legal representatives before the MRI procedure. The study was conducted at the Children’s Hospital 02 over a period of ten months, beginning in February 2019. In this study, all patients were enrolled and divided into two groups including group 1 with cerebellar medulloblastoma patients and group 2 with brainstem glioma patients. All medulloblastoma patients underwent either surgery or biopsy to obtain histopathological results, whereas all brainstem glioma patients were confirmed as brainstem glioma based on the full agreement between neuroradiologists and neurosurgeons [7,8].

### 2.2. Anesthesia Procedure

The patient was placed on the MRI table, in the supine position. The clinicians then performed the anesthesia procedure by injecting midazolam (5 mg/1 mL), at a dose of 0.1 mg/kg (Hameln Pharm GmbH, Hameln, Germany) and 1% intravenous anesthetic propofol (10 mg/1 mL), at a dose of 3 mg/kg (Fresofol, Fresenius Kabi GmbH, Graz, Austria).

### 2.3. MRI Procedure

In this study, all patients were scanned with a 1.5 Tesla MRI machine (Multiva, Philips, Best, The Netherlands) and assessed using the DWI sequence, with the following detailed parameters: repetition time (TR): shortest; echo time (TE): shortest; Flip angle: 90 degrees; Slice thickness: 5 mm; Gap: 1 mm; Field of view: 230 mm × 230 mm; Matrix: 144 mm × 90 mm; Plane: Axial; Number of Acquisitions: 2; b values: 0, 25, 50, 100, 200, 1000, and 1500; Duration: 3.43 min. ADC was automatically arisen from DWI, after finishing the scan.

### 2.4. Variables

**Figure 1:**
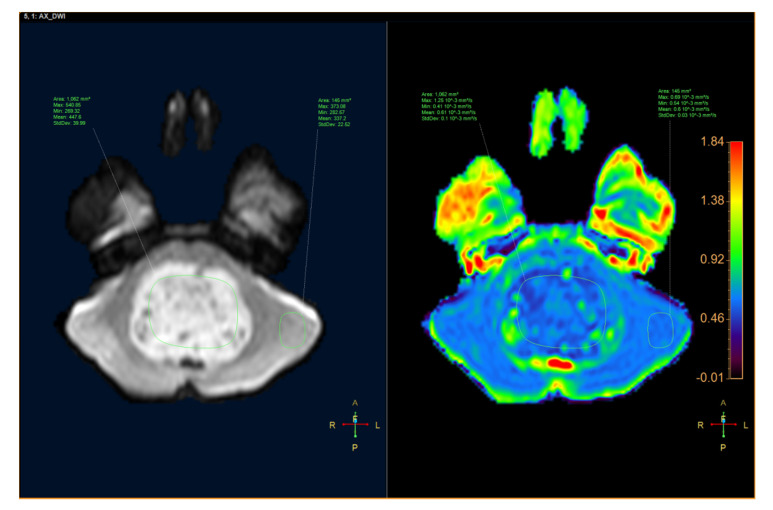
An 8-year-old male patient had a tumor inside the fourth ventricle, which was confirmed as medulloblastoma after surgery. (**Left**) Axial diffusion-weighted image (DWI). (**Right**) apparent diffusion coefficient (ADC) map.

**Figure 2:**
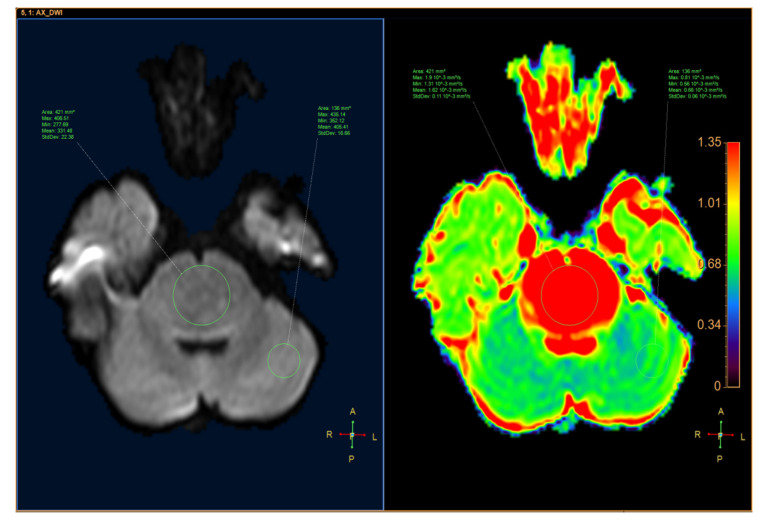
A 10-year-old male patient had a tumor located in the pons, which was diagnosed as a diffuse glioma. (**Left**) Axial DWI image. (**Right**) ADC map.

ADC was quantified by defining the region of interest (ROI) for the tumor and the parenchyma on the ADC map with MR Diffusion tool available in Philips Intellispace Portal, version 11 (Philips, Best, The Netherlands). ADC will provide the following parameters: maximum ADC (ADCmax), minimum ADC (ADCmin), mean ADC (ADCmean), and standard deviation ADC (ADCsd). The following additional parameters were also assessed: the ratio of tumor ADCmax to parenchyma ADCmax (rADCmax), the ratio of tumor ADCmin to parenchyma ADCmin (rADCmin), the ratio of tumor ADCmean to parenchyma ADCmean (rADCmean), and the ratio of tumor ADCsd to parenchyma ADCsd (rADCsd) (Figure 1 and Figure 2).

### 2.5. Statistics

SPSS software version 26 (IBM Corp, Armonk, New York, NY, USA) was used for statistical analysis. Quantitative variables are presented as the median and interquartile range. We compared quantitative variables with the Mann–Whitney U test. Receiver operating characteristic (ROC) curve analysis and the Youden index were used to evaluate the cut-off point, accuracy, sensitivity, and specificity. Differences were considered statistically significant when *p* < 0.05.

## 3. Results

In this study, 32 patients (median age: 7; male/female: 17/15) were enrolled and divided into two groups. Group 1 contained children with cerebellar medulloblastoma (*n* = 22, median age: 8; male/female: 13/9), and group 2 contained children with brainstem glioma (*n* = 10, median age: 6; male/female: 4/6).

As described in Table 1, ADCmax, ADCmin, ADCmean, ADCsd, rADCmax, rADCmin, rADCmean, and rADCsd values of medulloblastomas were 1.06, 0.43, 0.62, 0.11, 1.61, 0.80, 1.01, and 3.87, respectively, while those of brainstem gliomas were 1.64, 1.11, 1.39, 0.10, 1.96, 1.87, 2.05, and 2.25, respectively. The ADCmax, ADCmin, ADCmean, rADCmin, and rADCmean values for medulloblastomas were significantly lower than those for brainstem gliomas (*p* < 0.05).

**Figure 3:**
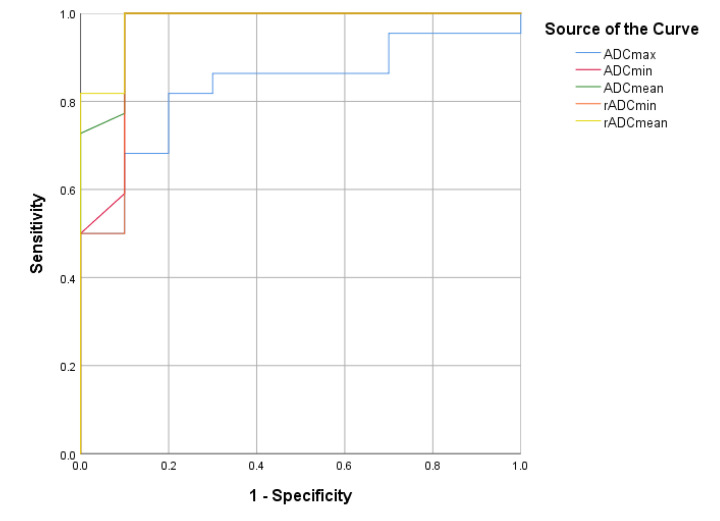
The ROC curves for the ADCmax, ADCmin, ADCmean, rADCmin, and rADCmean values.

As described in Table 2, among all parameters, a cut-off rADCmean value of 1.47 was better determined for the differential diagnosis between medulloblastomas and brainstem gliomas, which yielded the highest sensitivity value of 100%, a specificity of 90%, and an AUC of 98.2% (Figure 3).

## 4. Discussion

Our study found that the ADC values for medulloblastomas were significantly lower than those for brainstem gliomas. Significant differences were noted for ADCmax, ADCmin, ADCmean, rADCmean, and rADCmin values between medulloblastomas and brainstem gliomas. Among these parameters, a cut-off rADCmean value of 1.47 was shown to discriminate between medulloblastoma and brainstem glioma with a sensitivity of 100%, a specificity of 90%, and an AUC of 98.2%. Several other studies examining various ADC values have reported similar results [9,10,11,12,13,14,15,16,17,18].

Various ADC values have been suggested by different authors over the years to assist in the differentiation between medulloblastoma and brainstem glioma. Rumboldt et al. [9] suggest an ADC value of 0.66 ± 0.15 × 10^−3^ mm^2^/s for medulloblastoma, whereas Lober et al. [18] reported that the ADC of brainstem glioma was 1.295 × 10^−3^ mm^2^/s. Additionally, according to the study reported by Chen and colleagues [17] which examined 9 patients with brainstem glioma and 6 patients with medulloblastoma, the ADC of the brainstem glioma group was significantly higher than that of the medulloblastoma group (1.14 ± 0.18 × 10^−3^ mm^2^/s versus 0.56 ± 0.05 × 10^−3^ mm^2^/s, *p* < 0.001). In another study by Pierce et al. [16], the medulloblastoma group (*n* = 33) had an ADCmin of 0.54 × 10^−3^ mm^2^/s and the tumor ADC to parenchyma ADC ratio was 0.7, whereas the astrocytoma group (*n* = 50) had an ADCmin of 1.28 × 10^−3^ mm^2^/s and a tumor/parenchyma ADC ratio of 1.64. ROC analysis showed that a threshold ADCmin value of 0.66 × 10^−3^ mm^2^/s was the most significant measurement for distinguishing between medulloblastoma and other tumors. Mohamed and colleagues [12] found that in 7 medulloblastoma patients, the ADC index ranged from 0.5–0.9 × 10^−3^ mm^2^/s. The authors recommended that an ADC cut-off threshold of less than 0.9 × 10^−3^ mm^2^/s could be used to diagnose medulloblastoma. Jaremko et al. [10] found that an ADCmin value of 0.8 × 10^−3^ mm^2^/s could distinguish between medulloblastomas and low-grade astrocytomas. In general, the ADC values for brainstem glioma ranged from 1.14–1.29 × 10^−3^ mm^2^/s, which was significantly higher than the range of ADC values for medulloblastoma, which ranged from 0.5–0.9 × 10^−3^ mm^2^/s [9,10,12,16,17,18]. In our study, the median ADCmean and rADCmean values for medulloblastoma was 0.62 × 10^−3^ mm^2^/s and 1.01, respectively, whereas the median ADCmean and rADCmean values for brainstem glioma was 1.39 × 10^−3^ mm^2^/s and 2.05, respectively. Our findings are consistent with those reported by previous studies.

These differences can be explained by understanding the theory underlying DWI and ADC values. DWI calculates the Brownian movement of water molecules inside a voxel of tissue. Diffusion within any particular tissue is constrained by the borders formed by cell membranes, unlike the free motion of water that can occur in an unrestrained container. The total diffusion function of a single voxel reflects the amount of water diffusion that occurs in intracellular, extracellular, or both compartments within the tissue. The intracellular space includes the spaces within individual cells, which include the cytoplasm and organelles, whereas the extracellular space includes spaces within the intravascular, lymphatic, interstitial, and intracavitary regions. Any increases in the cellular density, odd substances, or heavy particles within those spaces will result in a reduction of the diffusion coefficient [8,9,10,11,12,16,19,20]. Medulloblastoma is a malignant brain tumor, characterized by high cell numbers and density [8,9,10,11,12,13,14,15,16,20,21,22,23]. The reduction in free water movement caused by narrow inter- and intracellular spaces will result in reduced signal production. Hence, the apparent diffusion velocity of medulloblastoma tumors will be low. In contrast, brainstem gliomas are less dense. The intercellular space is usually more spacious, such that hydrogen protons are not significantly restricted [8,9,10,11,12,13,14,15,16,19,20,22].

Our study has some limitations, which include small sample size and single-center involvement. Additionally, we did not examine morphological characteristics or perform whole ADC-histogram tumor analysis. In addition, the brainstem glioma patients were diagnosed clinically, with no histopathological examinations, due to our hospital guidelines. We would recommend that further studies, including larger sample sizes and multicenter involvement, be performed to validate our current findings.

## 5. Conclusions

The ADCmean ratio between the tumor and the parenchyma was the most effective parameter for differentiating between medulloblastoma and brainstem glioma, with an AUC of 98.2%. Other parameters that could also help clinicians differentiate between these two tumors include ADCmean, ADCmax, ADCmin, and rADCmin values. Further studies that include larger sample sizes and multicenter involvement should be performed to validate our current findings.

## Figures and Tables

**Table 1:** Comparison of the basic characteristics between medulloblastomas and brainstem gliomas.

	Medulloblastoma *n* = 22	Brainstem Glioma *n* = 10	*p*
**ADC**			
ADCmax (10^−3^ mm^2^/s)	1.06 (0.58)	1.64 (0.45)	0.003 ^§^
ADCmin (10^−3^ mm^2^/s)	0.43 (0.11)	1.11 (0.34)	<0.001 ^§^
ADCmean (10^−3^ mm^2^/s)	0.62 (0.19)	1.39 (0.33)	<0.001 ^§^
ADCsd	0.11 (0.06)	0.10 (0.05)	0.967
**ADC Ratio**			
rADCmax	1.61 (0.87)	1.96 (0.46)	0.067
rADCmin	0.80 (0.17)	1.87 (0.61)	<0.001 ^§^
rADCmean	1.01 (0.23)	2.05 (0.51)	<0.001 ^§^
rADCsd	3.87 (2.88)	2.25 (2.50)	0.185

^§^ Statistically significant.

**Table 2:** Receiver operating characteristic (ROC) analysis of ADC and rADC parameters for the differential diagnosis between medulloblastomas and brainstem gliomas.

	Cut-Off Point	AUC	Sensitivity	Specificity	95% CI
**ADC**					
ADCmax	1.45	0.832	0.818	0.8	0.688–0.975
ADCmin	0.68	0.955	1	0.9	0.865–1.000
ADCmean	0.95	0.975	1	0.9	0.922–1.000
**ADC Ratio**					
rADCmin	1.25	0.950	1	0.9	0.853–1.000
rADCmean	1.47	0.982	1	0.9	0.941–1.000
